# Detrimental alteration of mesenchymal stem cells by an articular inflammatory microenvironment results in deterioration of osteoarthritis

**DOI:** 10.1186/s12916-023-02923-6

**Published:** 2023-06-19

**Authors:** Mengqiang Fan, Peijian Tong, Li Yan, Ting Li, Jiadan Ren, Jiefeng Huang, Wenxi Du, Li Zhou, Letian Shan

**Affiliations:** 1 The First Affiliated Hospital, Zhejiang Chinese Medical University, Hangzhou, China; 2 Cell Resource Bank and Integrated Cell Preparation Center of Xiaoshan District, Hangzhou Regional Cell Preparation Center (Shangyu Biotechnology Co., Ltd), Hangzhou, China; 3 Department of Plastic & Reconstructive Surgery, Zhejiang Provincial People’s Hospital (Affiliated People’s Hospital, Hangzhou Medical College), Hangzhou, China; 4 College of Pharmaceutical Sciences, Zhejiang Chinese Medical University, Hangzhou, China

**Keywords:** Knee osteoarthritis, Synovial fluid, Human umbilical cord mesenchymal stem cells, Articular inflammatory microenvironment, MMP13, c-Jun, MAPKs

## Abstract

**Background:**

Articular injection of mesenchymal stem cells (MSCs) has been applied to treat knee osteoarthritis (kOA), but its clinical outcomes are controversial. This study investigated whether an articular inflammatory microenvironment (AIM) impacts MSC-based therapy in a rat model of kOA.

**Methods:**

The biological change of MSCs and the functional change of MSCs on chondrocytes were evaluated under AIM. The key mediator and mechanism for the AIM impact on MSC therapy were explored via gain- and loss-of-function approaches.

**Results:**

The results showed that MSCs exerted potent anti-kOA effects in vivo and in vitro, but that this therapy become chondrodestructive if a chronic AIM was present. Mechanistically, the overexpression of MMP13 in the injected MSCs via a MAPKs-AP1 signaling axis was revealed as the underlying mechanism for the detriment outcome.

**Conclusions:**

This study thus clarifies recent clinical findings while also suggesting a means to overcome any detrimental effects of MSC-based therapy while improving its efficacy.

**Supplementary Information:**

The online version contains supplementary material available at 10.1186/s12916-023-02923-6.

## Background

Knee osteoarthritis (kOA) is commonly occurred as a main cause of adult disability, with almost 30% of people over 45 years of age showing radiographic evidence of kOA, of which approximately half have symptoms, which accounting for 2.4% of all individuals with a disability [1, 2]. According to authoritative guidelines, weight loss, physical exercise, medication with nonsteroidal anti-inflammatory drugs (NSAIDs), and intra-articular injection of corticosteroid are recommended for nonsurgical treatment of kOA. However, none of these recommended approaches have achieved success in the cessation or reversal of kOA progression or long-term improvement of kOA symptoms [3, 4]. Moreover, some of them even cause adverse events, e.g.*,* increased cardiovascular risk and gastrointestinal complications with oral NSAID use [5–7]. Hence, arthroplasty has become the only viable option available for patients with kOA, resulting in a heavy financial burden on the healthcare system, with increased costs annually, but with unsatisfactory outcomes [8].

Intra-articular mesenchymal stem cells (MSCs) injection is a new developed therapy for kOA due to the pro-regenerative and repair capacity of these cells on damaged tissues [9–11]. In one study, MSC-based therapy substantially ameliorated kOA symptoms in patients as determined by increasing score (from 1.89 ± 0.3 to 2.91 ± 0.37) Western Ontario and McMaster Universities Osteoarthritis Index (WOMAC) and decreasing visual analog scale (VAS) score (from 57 ± 33 to 11.6 ± 24) at a 1-year follow-up [12]. However, although MSCs from many sources (bone marrow, adipose, umbilical cord, etc.) have exerted therapeutic potential in the clinic, the therapy remains controversial due to inconsistent outcomes [13–19]. It seems that MSC-based therapy has both beneficial and detrimental effects, as in some cases MSC-based therapy can ameliorate kOA, but in others, it actually worsened joint pain with no beneficial therapeutic outcome [20]. Such discrepancies indicate that the effectiveness of MSCs could be impacted by unknown variables, including differences in baseline conditions among patients [10, 16].

Synovitis is a typical manifestation of kOA caused by the effusion into the joint of proinflammatory synovial fluid (SF), resulting in palpable joint swelling and pain [21–25]. Among individuals with painful kOA, more than 40% had SF effusion, and the association between knee synovitis and SF effusion was highly significant (*P* < 0.0001) [26]. Synovitis is marked by an inflammatory microenvironment in the joint cavity [21–27]. As the microenvironment is a decisive factor for cell survival, we hypothesized that pre-existing synovitis may negatively impact the therapeutic efficacy of intra-articular MSC-based therapy. Notably, the proinflammatory SF contains a variety of inflammatory factors, such as interleukin-1β (IL-1β), interferon-gamma (IFN-γ), and tumor necrosis factor-α (TNF-α), which have been reported to negatively impact MSCs [28–32]. For instance, IL-1β promotes inflammation and can alter the immunosuppressive capacity of MSCs [33, 34], while IFN-γ inhibits the proliferation and differentiation of MSCs, and TNF-α negatively affects the survival, proliferation, and osteogenic differentiation of MSCs [35, 36]. Moreover, SF has been reported to alter the secretome of MSCs [37].

Given these effects, synovitis and an articular inflammatory microenvironment (AIM) might be the key reason behind the inconsistent outcomes of MSC-based therapy for kOA. The main goal of this study is to test the above hypothesis by determining the impact of AIM on MSCs in the treatment of kOA and exploring the underlying mechanism of AIM’s impact. By using a rat model of kOA, this study evaluated the therapeutic effects of MSCs on kOA and tested the impacts of AIM on MSCs by comparing the MSCs’ effects alone or in the presence of SF. The most commonly used animals for kOA modeling are rats and mice due to their low maintenance costs and relative ease of handling and genetic manipulation, making them more suitable for synthetic and genetically related primary kOA models [38]. In comparison with mice, rats have larger joint cavities and easier operability for articular injection, which may increase the success rate of operations in the kOA-related experiments. Moreover, rats can provide higher amounts of blood samples and bigger sizes of joint samples, which means that enough sample amounts and sizes would avoid excessive waste of animal numbers. Therefore, the rat model was chosen for this study. The results showed that matrix metalloproteinase 13 (MMP13) expression in MSCs was dramatically elevated in the presence of SF, leading to the deterioration of kOA in a rat model, which was marked by chondrodestruction and pain. The mechanism study elucidated that the MMP13 overexpression was due to the activation of MAPKs-AP1 signaling. Therefore, this study achieved the goal that the detrimental impact of AIM on MSCs was determined and the related mechanism was explored (Additional file 1: Fig. S1). It is known that MMP13 is normally excreted by abnormal chondrocytes (i.e., hypertrophic chondrocytes), and the overproduction of MMP13 in joint cavity is responsible for the degradation of cartilage matrix [39]. This study also pointed out that MSCs were another source of MMP13 which might deteriorate kOA.

## Methods

### Reagents and materials

Bicinchoninic acid (BCA) and 4′,6-diamidino-2-phenylindole (DAPI) were purchased from Thermo Fisher Scientific (MA, USA). Tris-buffered saline (TBS), low electrolyte bovine serum albumin (BSA)-fraction V, and phosphate-buffered saline (PBS) were purchased from Sangon Biotech (Shanghai, China). Nucleoplasmic separation kit was purchased from CWBIO (Beijing, China). X-tremeGENE HP DNA transfection reagent was purchased from Roche (Basel, Switzerland). INTERFERin SiRNA Transfection Reagent was purchased from Polyplus (Strasbourg, France). Cell culture plates were purchased from Eppendorf (Hamburg, Germany). Transwell (co-culture) chambers (3.0 μm pore size) were purchased from Corning (NY, USA). Minimum essential medium-alpha modification (α-MEM) with Glutamax™-1 was purchased from Gibco (NY, USA). Fetal bovine serum (FBS) was purchased from CellMax (Beijing, China). All-in-One cDNA Synthesis SuperMix kit was purchased from Biotool (TX, USA). 2 × SYBR Green qPCR Master Mix (low ROX) kit, protease inhibitor cocktail and phosphatase inhibitor cocktail were purchased from Bimake (TX, USA). Nitrocellulose membrane was purchased from Sartorius Stedim (Göttingen, Germany). Immunohistochemistry (IHC) kits were purchased from ZSGQ-BIO (Beijing, China). siRNA oligos were purchased from RIBOBIO (Guangzhou, China). All antagonists were purchased from Selleck (Houston, USA). Details of antibodies used in this study are presented in Additional file 2: Table S1.

### Inclusion and exclusion of kOA patients

The inclusion criteria were as follows: (1) aged 30 to 80 years; (2) confirmed kOA diagnosis by radiological and clinical evaluations; (3) Kellgren-Lawrence grade 2 or above; and (4) first-visit outpatients without previous kOA medication history. The exclusion criteria were as follows: (1) combined with other types of arthritis, such as rheumatoid arthritis; (2) combined with other disease, such as malignant tumor, liver failure, kidney failure, and cardiovascular diseases; (3) active or severe infections; and (4) intake of anti-inflammatory drugs in the past 2 weeks. This study was approved by the Ethics Committee of the First Affiliated Hospital of Zhejiang Chinese Medical University (Ethical number: 2020-KL-151–01).

### Collection and cytokine analysis of SF from kOA patients

About 10 ml of SF was obtained by needle aspiration from each kOA patient (*n* = 23) before any therapeutic operations (ethical approved). The informed consent of each voluntary patient was obtained before the operation. Each sample was centrifuged at 14,000 × *g* for 20 min and residual impurities were removed. An equal amount of supernatant from each patient was mixed and stored at − 80 °C until use. Luminex analysis was performed using 3 ml of the mixed SF to detect cytokines and their concentrations. Luminex instrument (X-200, Luminex, Texas, USA) was utilized and the cytokines in SF were identified and quantified [40, 41].

### Isolation and culture of primary cells

The primary hucMSCs (P3) were provided by the Cell Resource Bank and Integrated Cell Preparation Center of Xiaoshan District, Hangzhou Regional Cell Preparation Center. The cells were seeded in α-MEM with Glutamax™-1 containing 10% FBS at 37 °C and 5% CO_2_, and the culture medium was replaced every 2 days. For surface maker identification, the primary cells (10^6^ cells/ml) were conjugated with the following antibodies: CD34-PE (phycoerythrin), CD45-PE, CD73-PE, CD90-PE, CD105-PE, and HLA-DR-PE. The labeled cells were analyzed via flow cytometer (BD Accuri C6, NJ, USA). The identified hucMSCs at the logarithmic growth phase were used for assays.

Rat primary chondrocytes were isolated from the articular cartilage of healthy SD rats (Grade SPF II) donors. The sampling method was permitted by the Ethics Committee of Zhejiang Chinese Medical University (Ethical number: 20190506–14). Fresh cartilage tissues were dispersed in 0.25% trypsin (Thermo Fisher Scientific, MA, USA) and then treated with 0.1% collagenase II (Worthington, OH, USA). The detached chondrocytes were collected and cultured in Iscove’s modified Dulbecco's medium (IMDM, Thermo Fisher Scientific, MA, USA) containing 10% FBS. The chondrocytes at passage 3 to 5 were used for in vitro assays.

### Animals and kOA modeling

Male SD rats (Grade SPF II) weighing 200 ± 20 g (8 weeks old) were provided by Shanghai Super B&K Laboratory Animal Co. Ltd. (Certificate number: SCXK (Shanghai) 2018–0006). All animal experiments were carried out in accordance with the guidelines of the Committee for the Purpose of Control and Supervision of Experiments on Animals (CPCSEA) and approved by the Ethics Committee of Zhejiang Chinese Medical University (Ethical number: 20190506–14). All rats were housed in polypropylene cages (5 per cage) and maintained at 22 ± 1 °C with 12 h dark/light cycles and humidity of 50 ~ 60% with free access to food and water in an SPF condition. To minimize potential confounders, the cages were numbered and positioned in a preassigned order, and the same operators performed the subsequent treatments and measurements in that order. After 1-week acclimatization, an anterior cruciate ligament transection (ACLT) kOA model was established after anesthesia as described previously [42]. Anterior drawer test was applied to check the success of modeling.

### In vivo efficacy evaluation of hucMSCs in the presence/absence of AIM

To mimic the clinical situation of AIM, the kOA rat model was employed to conduct an animal experiment (Additional file 3: Fig. S2). The kOA patients-obtained SF samples were stored at − 80 °C and used for intra-articular injection. A total of 50 rats (5 per cage) were randomly divided into 5 groups by dice method (*n* = 10): the normal control (NC) group and the model group received articular injections of PBS solution (50 μl), the MSC-treatment group received an articular injection of a hucMSCs suspension (10^6^ cells/ml) in PBS solution (50 μl), the SF group received an articular injection of 50 μl SF, and the MSC + SF group received an articular injection of a hucMSCs suspension (10^6^ cells/ml) in SF. The rats successfully modeled by ACLT (*n* = 40) were included in all groups except the NC group, and the total number of rats and the number of rats in each group were determined according to the Resource Equation Approach [43]. There were no exclusions of animals in each group. After the 8-week modeling, the injections were given once a week for 4 weeks. For the following tests, eight data points were obtained from eight rats randomly selected from each group. Data points from rats that died abnormally were excluded. The author who designed the experiment was aware of the group allocation at the different stages of the experiment. The authors who conducted the modeling and treatment experiments were also aware of the group allocation. However, the authors who obtained the outcomes and conducted the data analysis were not aware of the group allocation.

At 1 week after the final treatment, the mechanical withdrawal threshold (MWT) and thermal withdrawal latency (TWL) were measured for pain evaluation by von Frey filaments (range from 0.6 to 26 g, Ugo Basile, Lombardy, Italy) and a plantar test apparatus (Ugo Basile, Lombardy, Italy), respectively. Briefly, rats were individually placed into mechanical and thermal testing chambers and acclimated for 30 min without disturbance. For MWT assessment, von Frey filaments were pressed perpendicularly against the mid-plantar surface of the hind paws of each rat and held for at least 2 s at least 3 times. A positive response for each test was defined as the sharp withdrawal of the hind paws, and the maximal bending forces (g) of the filaments were recorded. For the assessment of TWL, a pain threshold detector was used to thermally stimulate the hind paws with light, and the light-shining was stopped at the appearance of paw lifting. TWL was defined as the shortest lighting duration. All measurements were conducted thrice at 4 min intervals to obtain the mean value.

The entire knee joints were dissected, and fixed in 4% paraformaldehyde (PFA) buffered for 72 h (4 °C). The samples were decalcified for 8 weeks with 10% ethylene-diamine tetra acetic acid (EDTA) (pH 7.4) at 4 °C. Each sample was embedded in paraffin and 3 μm sections were cut, followed by staining with SO (Safranin O/Fast green). A blinding method was utilized, in which three experimenters applied the Osteoarthritis Research Society International (OARSI) scoring system to observe and score the histopathological changes among different groups.

IHC was utilized to detect the expression of MMP13 and Col2. Replicates of each sample underwent antigen retrieval by incubation with 0.01 mol/l citrate buffer (pH 6.0, Solarbio, Beijing, China) at 60 °C for 4 h. The sections were incubated with the primary antibody of MMP13 and Col2 at 4 °C overnight, followed by incubation with secondary antibody at room temperature for 20 min. After washing 3 times, colorimetric visualization with diaminobenzidine solution (Invitrogen, MD, USA) was performed. The expression of MMP13 and Col2 was quantified by Image-Pro Plus 6.0 software (Media Cybernetics, MD, USA) under a light microscope (NIKON 80i, Tokyo, Japan). The data are presented as the pixel percentage of antigen-positive cells to total cells (MMP13) or antigen-positive area to total area (Col2) in 5 random fields.

### In vitro determination of the impact of AIM on hucMSCs

To evaluate the in vitro impact of AIM on the state of hucMSCs, hucMSCs were treated with SF and divided into 2 groups: NC as the normal hucMSCs group and SF as the SF-treated hucMSCs group (Additional file 4: Fig. S3). The medium of the SF group was replaced with α-MEM containing 10% FBS and 10% SF (v/v), while the medium of the NC group was replaced with α-MEM containing 10% FBS and 10% PBS (v/v). After co-culture for 48 h, hucMSCs in these groups were washed with PBS 3 times to remove the residual medium.

To evaluate the in vitro impact of AIM on the function of hucMSCs, a nested co-culture system containing hucMSCs (upper chamber) and chondrocytes (lower chamber) was employed and 4 groups were set as follows: an NC group containing untreated chondrocytes, an MSC group containing hucMSCs (3 × 10^5^ cells per well)-treated chondrocytes in the absence of SF, an SF group containing SF-treated chondrocytes, and an MSC + SF group containing hucMSCs-treated chondrocytes in the presence of SF (Additional file 5: Fig. S4). The cells in each chamber were seeded at a density of 3 × 10^5^ cells. The medium of the NC group and the MSC group was IMDM containing 10% FBS and 10% PBS (v/v), while the medium of the SF and the MSC + SF group was IMDM containing 10% FBS and 10% SF (v/v).

### Immunofluorescence

After nested co-culturing for 48 h, the cells on coverslip were fixed with 4% PFA and permeabilized with 0.1% Triton X-100 in PBS. Thereafter, the cells were incubated with primary PCNA (proliferating cell nuclear antigen) antibody in 1% BSA at 4 °C overnight and followed by treatment with a secondary antibody. The nuclei were stained by using 10 ng/ml DAPI.

### Wound healing assay

Before nested co-culturing, the chondrocytes layer was scratched using a sterile 200 μl pipette tip and washed with PBS to remove detached cells. Subsequently, the cells were observed and imaged under an inverted microscope (Carl Zeiss, Göttingen, Germany) at 0, 24, and 48 h. The wound area was calculated by Image J 1.47 software as follows: blank area ratio (%) = Ar/At × 100%, where “At” represented the total area in view, and “Ar” represented the blank area of the wound.

### Gene overexpression and knockdown

To explore whether MMP13 played a determinant role in the impact of AIM on hucMSCs, an MMP13-overexpression (MMP13^OE^) plasmid was constructed by Genechem Co., Ltd. (Shanghai, China). For MMP13 overexpression, MMP13^OE^ plasmid were delivered by X-tremeGENE HP DNA transfection reagent at a ratio of 1:2 according to the manufacturer’s instructions.siRNAs were utilized to transiently knockdown targeted genes (MMP13, c-Jun, c-Fos, and p65). The sequences of siRNA were listed in Additional file 6: Table S2. siRNAs or scrambled siRNA (si NC) were mixed with INTERFERin SiRNA Transfection Reagent (1:2.5) at room temperature for 15 min. Then, these siRNAs were delivered to hucMSCs following the manufacturer’s instructions.

### Quantitative real-time PCR (qRT-PCR)

The relative mRNA expressions of targeted genes in hucMSCs or chondrocytes were analyzed by qRT-PCR assay on an ABI QuantStudioTM 7 Flex Real-Time PCR System (Thermo Fisher Scientific, MA, USA) using SYBR® Premix Ex Taq II (Tli RnaseH Plus) kit. The sequences of primers are presented in Table 1.

**Table 1:** Primer sequences of targeted genes of human (h) and rat (r)

Gene	Forward primer	Reverse primer
*h-β-ACTIN*	5′-CCCGCGAGTACAACCTTCT-3′	5′-CGTCATCCATGGCGAACT-3′
*h-MMP13*	5′-CCAGACTTCACGATGGCATTG-3′	5′-GGCATCTCCTCCATAATTTGGC-3′
*r-β-actin*	5′-CCCGCGAGTACAACCTTCT-3′	5′-CCCGCGAGTACAACCTTCT-3′
*r-Col2*	5′-CTCAAGTCGCTGAACAACCA-3′	5′-GTCTCCGCTCTTCCACTCTG-3′
*r-Sox9*	5′-CATCAAGACGGAGCAACTGA-3′	5′-TGTAGTGCGGAAGGTTGAAG-3′
*r-Adamts4*	5′-TTCGCTGAGTAGATTCGTGGAG-3′	5′-CGGACTTTTATGTGGGTTGC-3′
*r-Adamts5*	5′-TGGAGTGTGTGGAGGGGATA-3′	5′-CGGACTTTTATGTGGGTTGC-3′
*r-Mmp13*	5′-CTATGGTCCAGGAGATGAAGAC-3′	5′-GTGCAGACGCCAGAAGAATCT-3′
*r-Col10*	5′-GATCATGGAGCTCACGGAAAA-3′	5′-CCGTTCGATTCCGCATTG-3′

### Western blot (WB)

The total proteins of hucMSCs and chondrocytes were extracted and analyzed by WB as previously described [44–46]. To separately analyze the transcriptional factors (p65, c-Jun, c-Fos, and FosB) in the cell nucleus and cytoplasm, a nucleoplasmic separation kit was utilized to separate the nuclear and cytoplasmic proteins according to the manufacturer’s instructions. The protein concentration was determined by the BCA method, followed by electrophoresis and membrane transferring. After incubation with antibodies, each protein was visualized using Western Lightning® Plus ECL (Perkin Elmer, Inc., MA, USA) and detected using X-ray film (Kodak, Tokyo, Japan). Image J (version 1.8.0) software (National Institutes of Health, MD, USA) was used to statistically analyze the intensity of each protein band.

### Determination of the in vitro and in vivo role of MMP13 in hucMSCs under an AIM

In vitro, the nested co-culture system containing hucMSCs and chondrocytes were utilized to evaluate the effects of the *MMP13*-overexpressing hucMSCs (MSC^OE^) and the *MMP13*-knockdown hucMSCs (MSC^KD^) (Additional file 7: Fig. S5). In vivo, a total of 60 rats (5 per cage) were randomly divided into 6 groups by dice method (*n* = 10): an NC group containing normal rats, a model group containing kOA model rats, an MSC group containing kOA rats treated with normal hucMSCs, an MSC + SF group containing kOA rats treated with both normal hucMSCs and SF, an MSC^OE^ group containing kOA rats treated with MSC^OE^, and an MSC^KD^ + SF group containing kOA rats treated with MSC^KD^ and SF (Additional file 8: Fig. S6). The NC and the model groups were injected intra-articularly with a PBS solution (50 μl), the MSC and MSC^OE^ groups were injected with the corresponding hucMSCs at 10^6^ cells/ml in PBS solution (50 μl), and the MSC + SF and MSC^KD^ + SF groups were injected with the corresponding hucMSCs at 10^6^ cells/ml in SF (50 μl). The rats successfully modeled by ACLT (*n* = 50) were included in all groups except the NC group, and the total number of rats and the number of rats in each group were determined according to the Resource Equation Approach [43]. There were no exclusions of animals in each group. After the 8-week modeling, the injections were performed once a week for 4 weeks. The pain behavior tests, histopathological observation, and immunohistochemical analysis were conducted, and eight data points were obtained as described above.

### Mechanism behind the MMP13-mediated alteration of hucMSCs under an AIM

As MMP13 played a pivotal role in determining the detrimental effects of hucMSCs under an AIM, the underlying mechanism was explored by analyzing the upstream transcriptional factors and signaling molecules for MMP13 in hucMSCs. HucMSCs were seeded in 10 cm dishes (5 × 10^5^ cells per dish) and divided into 2 groups: an NC group injected with untreated hucMSCs and an SF group treated with SF-exposed hucMSCs. After 48 h of SF exposure, the cells were washed with PBS 3 times and collected for WB assay. Total proteins of the cell nucleus and cytoplasm were separately extracted to analyze the expressions of transcriptional factors (p65, c-Jun, c-Fos, and FosB) that regulate MMP13 expression, followed by siRNA interference on these factors for validation.

As c-Jun was revealed as the key factor for MMP13 in regulation hucMSCs, the nested co-culture system was applied to further confirm the role of a c-Jun-MMP13 axis in hucMSCs in treating chondrocytes. siRNA of c-Jun (si c-Jun) was utilized and 6 groups were set as follows: an si NC group containing si NC-transfected hucMSCs, a si c-Jun1 group containing si c-Jun1-transfected hucMSCs, an si c-Jun2 group containing si c-Jun2-transfected hucMSCs, an si NC + SF group containing si NC-transfected hucMSCs in the presence of SF, a si c-Jun1 + SF group containing si c-Jun1-transfected hucMSCs in the presence of SF, and a si c-Jun2 + SF group containing si c-Jun2-transfected hucMSCs in the presence of SF. After 48 h of co-culturing, chondrocytes in the lower chamber of each group were collected for WB analyses for markers of anabolism (namely, Col2 and Sox9) and of catabolism (MMP13). Regarding the upstream signaling for c-Jun-MMP13, MAPKs pathway-related molecules (Erk1/2, p38, and JNK) were analyzed by WB in the presence or absence of SF. Moreover, inhibitors of Erk1/2, p38, and JNK were utilized to validate the role of each molecule in regulating c-Jun and MMP13.

### Statistical analysis

Data were expressed as mean ± SD. Data from different groups were compared using one-way ANOVA followed by Fisher’s least significant difference (LSD) multiple comparison. A *P*-value < 0.05 was considered to indicate a significant difference, and *P*-value < 0.01 considered to indicate a very significant difference. All analyses were performed using an updated version of the Data Processing System (DPS) software (9.5) [47].

## Results

### SF from kOA donors

The joint SF were sampled from 23 outpatients (male: female = 9:14) of first visit (aged from 47 to 61 years) and mixed. The VAS scores ranged from 2 to 5 and the WOMAC scores ranged from 65 to 124 (Table 2). In the mixed SF from patients with kOA, many inflammatory factors (e.g., IL-1α, IL-6, IL-1β, IL-17, IL-12, IL-8, TNFα, TNFβ, and IFNγ) were detected (Additional file 9: Table S3).

**Table 2:** Information of SF donors

	Age (years)	VAS score	WOMAC score
Male (*n* = 9)	54.67 ± 4.64	3.67 ± 0.94	88.67 ± 13.64
Female (*n* = 14)	52.57 ± 3.81	4.07 ± 0.74	94.79 ± 17.33

### HucMSCs treatment leads to deteriorated kOA progression under an AIM

**Fig. 1:**
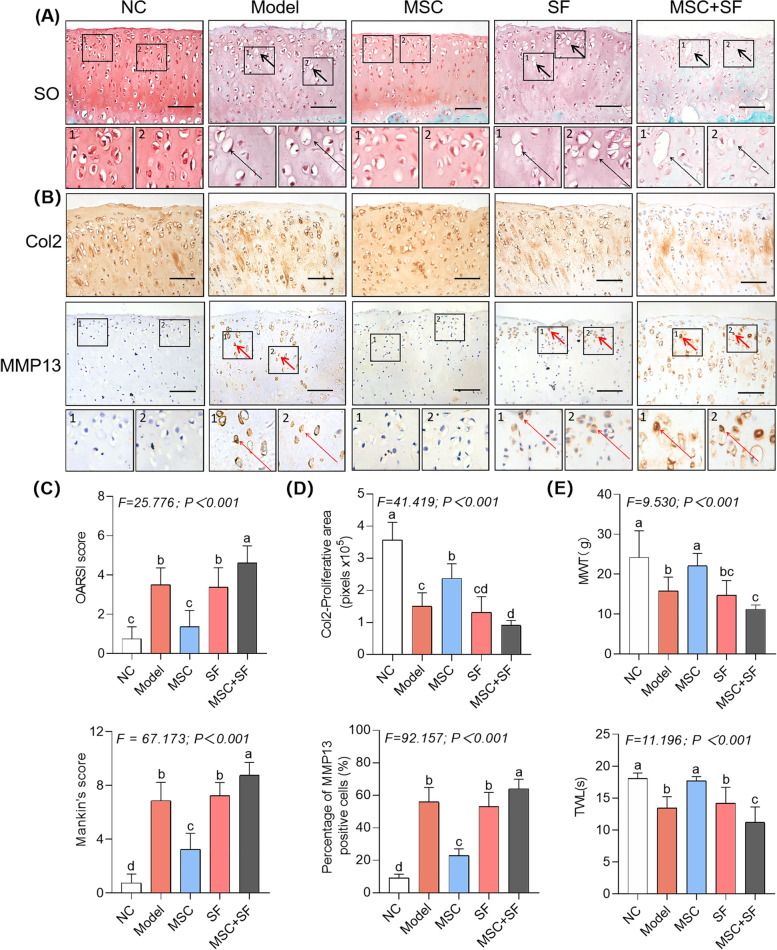
Histopathological observation, IHC analysis, and pain behavior tests on the in vivo effects of hucMSCs, SF, and SF-stimulated hucMSCs on kOA rats. **A** Safranin O/Fast green staining on cartilage with black arrows (hypertrophy or loss of chondrocytes). Scale bars = 50 μm. **B** IHC staining of Col2 and MMP13 on cartilage with red arrows (positive cells). Scale bars = 50 μm. **C** OARSI and Mankin’s scoring of histopathology. **D** Quantitative measurements of the percentage of Col2-positive area and MMP13-positive cells. **E** Measurement of MWT and TWL of rats. Data expressed as mean ± SD. The different letter symbols (a, b, bc, c, cd, and d) indicate significant difference between each other (Fisher’s LSD, *P* < 0.05 or *P* < 0.01) in the descending order of data from a to z. Although the overlapped letters (b versus bc, bc versus c, c versus cd, and cd versus d) were statistically different, their difference between each other was not significant. All experiments were repeated at least three times

By applying the ACLT kOA model, the in vivo effect of hucMSCs in the presence and absence of SF was evaluated. The hucMSCs were identified by flow cytometry (Additional file 10: Fig. S7). As shown in Fig. 1A, the typical kOA cartilage degeneration, such as glycosaminoglycan loss and chondrocyte hypertrophy, was observed in the model group, while chondroprotection against degeneration was clearly seen in the MSC group, demonstrating the anti-kOA efficacy of hucMSCs. However, a dramatic destruction of cartilage occurred in the MSC + SF group, which was even worse than that of the model group or the SF group, indicating that hucMSCs in the presence of SF worsened the cartilage degeneration. The histopathological scoring (Mankin’s score and OARSI score) indicated a consistent tendency of significant increasing in the following order: NC < MSC < Model ≈ SF < MSC + SF (Fig. 1C).

The IHC results exhibited significant abnormalities of anabolism (decreased expression of Col2) and catabolism (overexpression of MMP13) in the cartilage of the model group (*P* < 0.01 versus NC), which were significantly recovered in the MSC group (*P* < 0.01 versus Model), verifying the chondroprotective efficacy of hucMSCs (Fig. 1B and D). Moreover, the expressions of Col2 and MMP13 in the SF group were similar to that in the model group (*P* > 0.05), while their abnormalities were much worse in the MSC + SF group than either the model group or the SF group (each *P* < 0.01), confirming that hucMSCs in the presence of SF resulted in destruction rather than protection of the cartilage.

The pain behavior tests showed a similar tendency, in which the mechanical pain (MWT) and thermal pain (TWL) parameters were decreased significantly in the following order: NC > MSC > Model ≈ SF > MSC + SF (Fig. 1E). These results indicate that normal hucMSCs significantly restored the mechanical and thermal pain parameters in the MSC group, but the hucMSCs in the presence of SF not only lost the analgesia effect but also aggravated the pain in the MSC + SF group.

### HucMSCs damage chondrocytes under an AIM

**Fig. 2:**
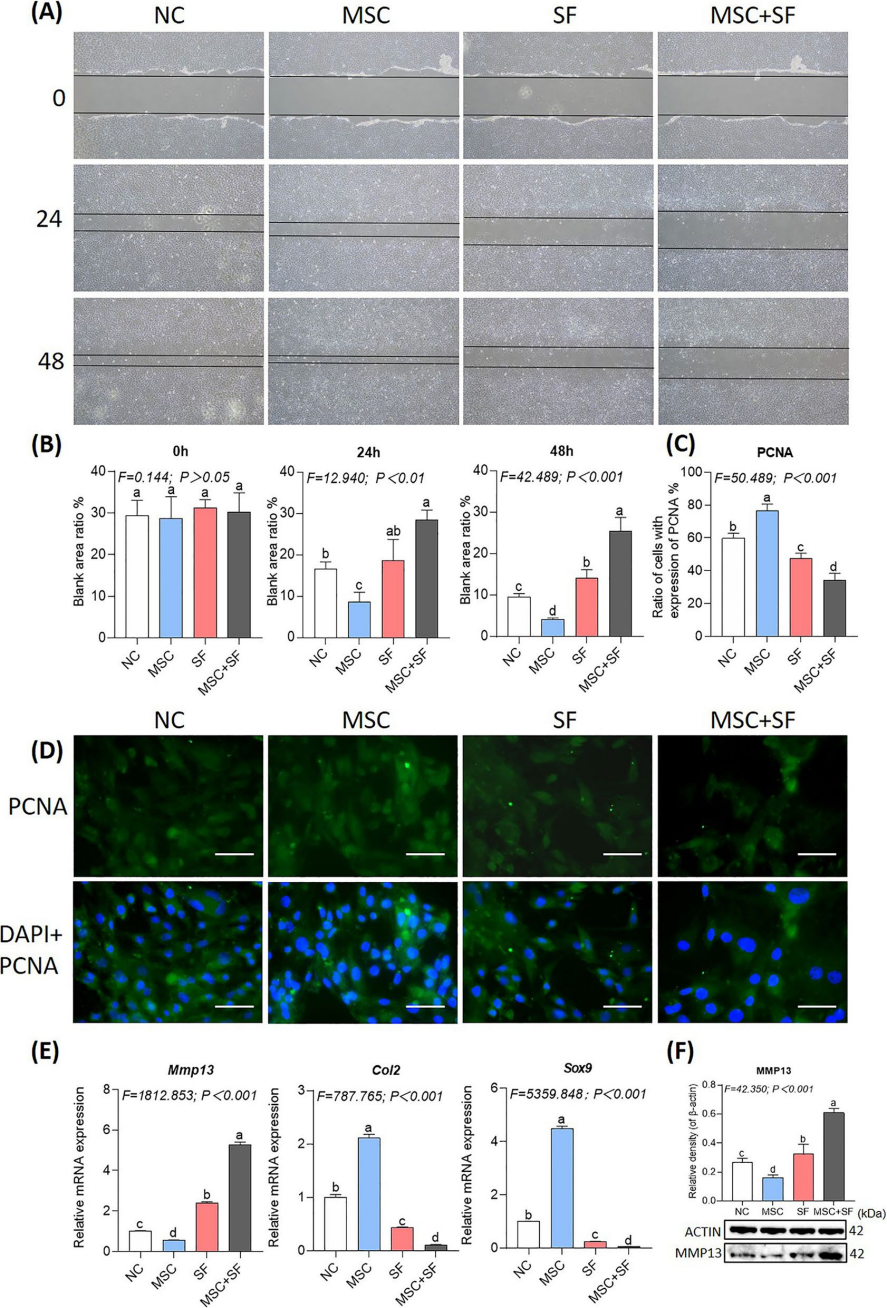
Evaluation of the wound healing and proliferation-regulatory effects of hucMSCs on chondrocytes before and after SF stimulation. **A** Wound healing of chondrocytes with hucMSCs and/or SF treatment at 0 h, 24 h, and 48 h. Scale bars = 200 μm. **B** Quantification of the wound area ratio (blank area/total area) at 0 h, 24 h, and 48 h. **C** Quantification of PCNA-expressed cell ratio (positive cells/total cells). **D** Cell immunofluorescence of chondrocytes with hucMSCs and/or SF treatment at 24 h, n = 3. **E** Expressions of anabolic and catabolic genes of chondrocytes. **F** Protein expression of MMP13 in chondrocytes. Values are presented as mean ± SD. The different letter symbols (a, ab, b, c, and d) indicate significant difference between each other (Fisher*'*s LSD, *P* < 0.05 or *P* < 0.01) in descending order of data from a to z. Although the overlapped letters (a versus ab and ab versus b) were statistically different, their difference between each other was not significant. All experiments were repeated at least three times

To further confirm the detrimental effects of hucMSCs in the presence of SF, a nested system was utilized to assess wound healing, cell immunofluorescence, qRT-PCR, and WB on rat chondrocytes. As shown in Fig. 2A and B, the wound ratio (blank area/total area) of chondrocytes in the MSC group was significantly decreased (each *P* < 0.01 versus NC) at 24 h and 48 h. Inversely, the ratio was significantly increased in the MSC + SF group (each *P* < 0.01 versus NC) and was higher than that in the SF group (each *P* < 0.01 versus SF) at 24 h and 48 h. As shown in Fig. 2C and D, the PCNA expression in chondrocytes was upregulated significantly in the MSC group (*P* < 0.01 versus NC) and was downregulated in the SF and MSC + SF groups in a descending order (each *P* < 0.01 versus NC). As shown in Fig. 2E and F, the gene expressions of Col2 and Sox9 were upregulated significantly in the MSC group (*P* < 0.01 versus NC) and downregulated in the SF and MSC + SF groups in a descending order (each *P* < 0.01 versus NC). Moreover, the gene and protein expression levels of MMP13 were downregulated significantly in the MSC group (*P* < 0.01 versus NC) and upregulated in the SF group and MSC + SF group in an ascending order (each *P* < 0.01 versus NC). The results demonstrate that hucMSCs in the absence of SF have beneficial effects on chondrocytes by promoting wound closure and cellular proliferation while improving anabolism versus catabolism at the molecular level, whereas hucMSCs in the presence of SF can damage chondrocytes by opposite actions.

### MMP13 overexpression occurs in hucMSCs under an AIM

**Fig. 3:**
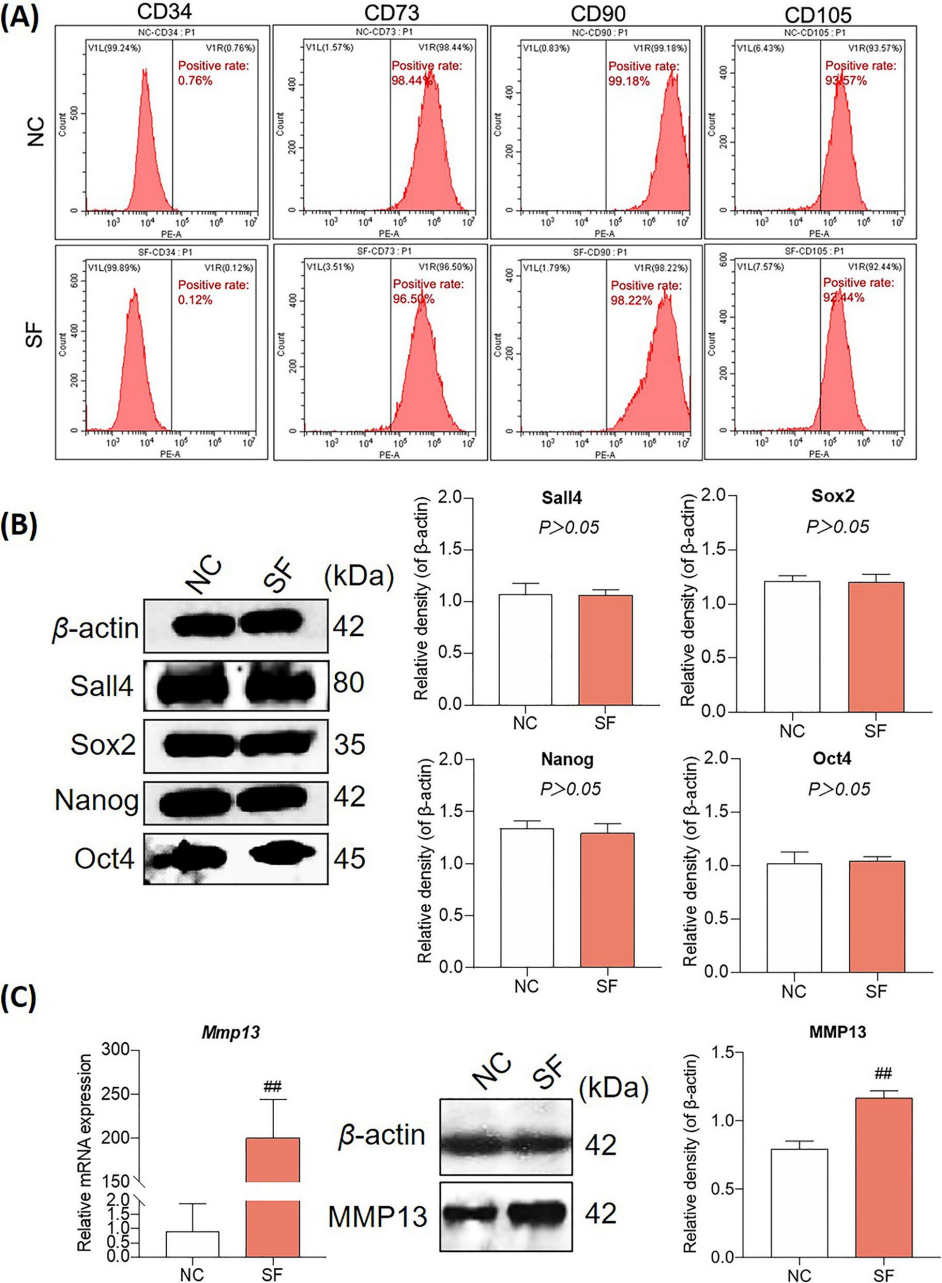
Evaluation of stemness and MMP13 expression of hucMSCs before and after SF stimulation. **A** Flow cytometry of the immunophenotype of mesenchymal stem cell surface markers of hucMSCs. **B** A WB of stemness-related proteins from hucMSCs. **C** qPCR and WB analyses of MMP13 expression in hucMSCs. Values are presented as mean ± SD. ^#^*P* < 0.05; ^##^*P* < 0.01. All experiments were repeated at least three times

As elucidated by flow cytometry and WB, both the MSC surface markers (CD73, CD90, CD105, and CD34) and stemness-related proteins (Sall4, Sox2, Nanog, and Oct4) were rarely changed in hucMSCs after SF stimulation, indicating little impact of AIM on the stemness and differentiation of hucMSCs (Fig. 3A and B). However, qRT-PCR and WB analyses revealed significant overexpression on the gene and protein levels of MMP13 in hucMSCs in the presence of SF (Fig. 3C). As MMP13 is a major catabolic enzyme that contributes to the development of kOA, the negative impact of AIM on hucMSCs-based therapy for kOA might be due to the overexpression of MMP13.

### MMP13 determines the impact of AIM on hucMSCs

**Fig. 4:**
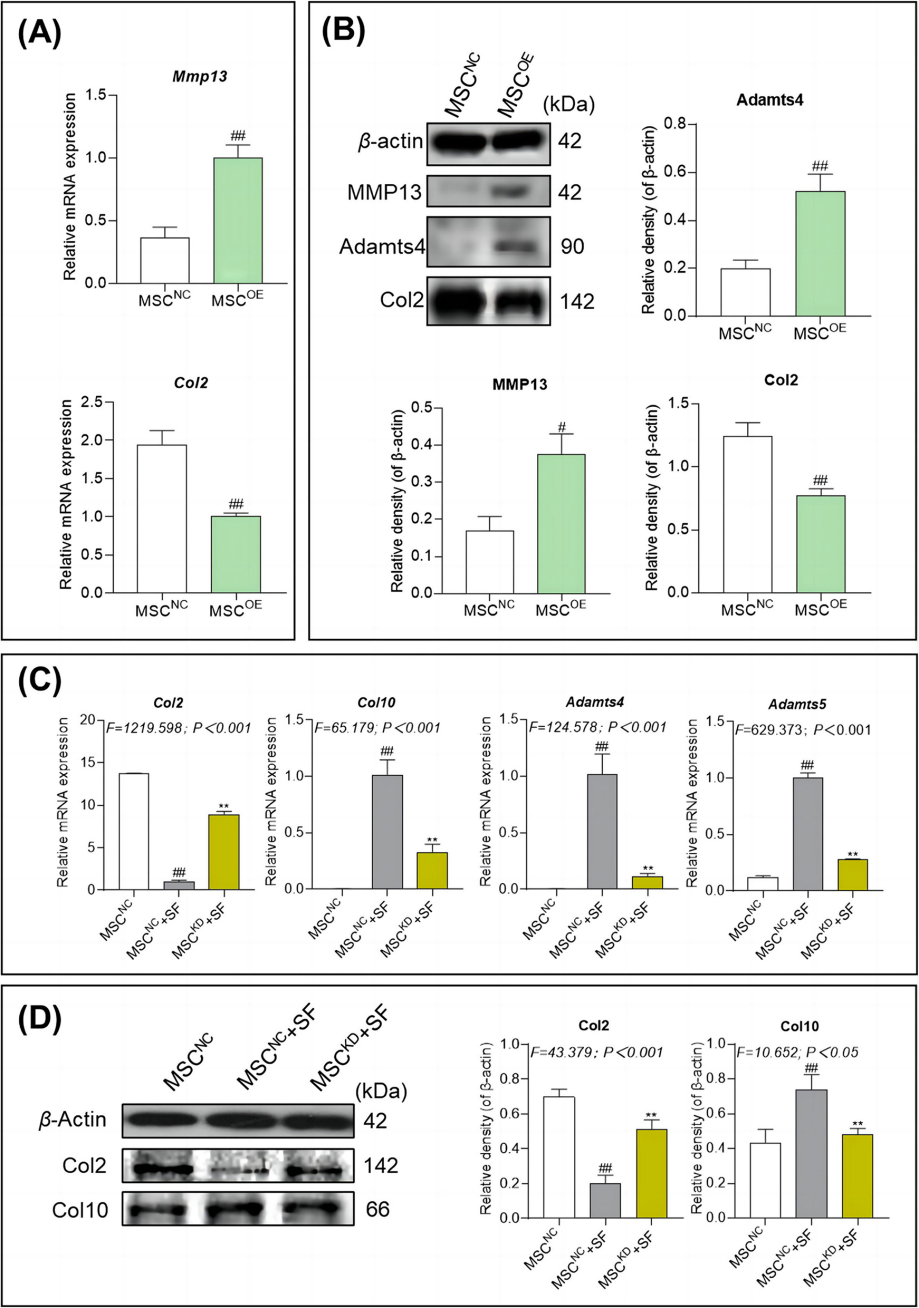
Evaluation of the anabolic and catabolic effects of MSC^OE^ and MSC^KD^ on chondrocytes by qRT-PCR and WB. **A** mRNA expression of *Mmp13* and *Col2* in chondrocytes treated with normal hucMSCs and *MMP13*-overexpressing hucMSCs. **B** Protein expression levels of MMP13, Adamts4, and Col2 in chondrocytes treated with normal hucMSCs or *MMP13*-overexpressing hucMSCs. **C** mRNA expression levels of Col2, Col10, Adamts4, and Adamts5 in chondrocytes treated with normal hucMSCs, normal hucMSCs in the presence of SF and *MMP13*-knockdown hucMSCs in the presence of SF. **D** Protein expression levels of Col2 and Col10 in chondrocytes treated with hucMSCs, hucMSCs in the presence of SF, and hucMSCs with *MMP13* knockdown in the presence of SF. MSC^NC^: nontargeting control siRNA-treated hucMSCs, MSC^KD^: MMP13-knockdown siRNA-treated hucMSCs. The medium of MSC^NC^ group was replaced by IMDM containing 10% FBS (v/v) and 10% PBS (v/v), and the medium of MSC^NC^ + SF group and MSC^KD^ + SF groups were replaced by IMDM containing 10% FBS (v/v) and 10% SF (v/v). Values are presented as mean ± SD. ^#^*P* < 0.05 versus MSC^NC^ group; ^##^*P* < 0.01 versus MSC^NC^ group; ^**^P < 0.01 versus MSC^NC^ + SF group. All experiments were repeated at least three times

MSC^OE^ and MSC^KD^ were utilized to validate the role of MMP13 on the detrimental effects of hucMSCs. As illustrated in Fig. 4A and B, the mRNA and protein expressions of catabolic markers (Mmp13 and Adamts4) were upregulated significantly and that of an anabolic marker (Col2) were downregulated in the MSC^OE^-treated chondrocytes (each *P* < 0.01 versus MSC^NC^ group). As illustrated in Fig. 4C and D, MSC^KD^ significantly inhibited the expressions of catabolic markers (Adamts4, Adamts5, and Col10) and significantly upregulated that of anabolic marker (Col2) in chondrocytes even in the presence of SF (each *P* < 0.01 versus MSC^NC^ + SF group), and the expressions of these markers attained the levels of the normal hucMSC-treated chondrocytes (each *P* > 0.05 versus MSC^NC^ group). The above results indicate that MMP13 overexpression is a key switch that determines the effects of hucMSCs-based therapy. When MMP13 levels are low (i.e., when SF is absent and the microenvironment is relatively non-inflammatory), the cells show potent anti-kOA efficacy, but when SF is present and there is an AIM, the hucMSCs actually have detrimental effects on kOA progression.

**Fig. 5:**
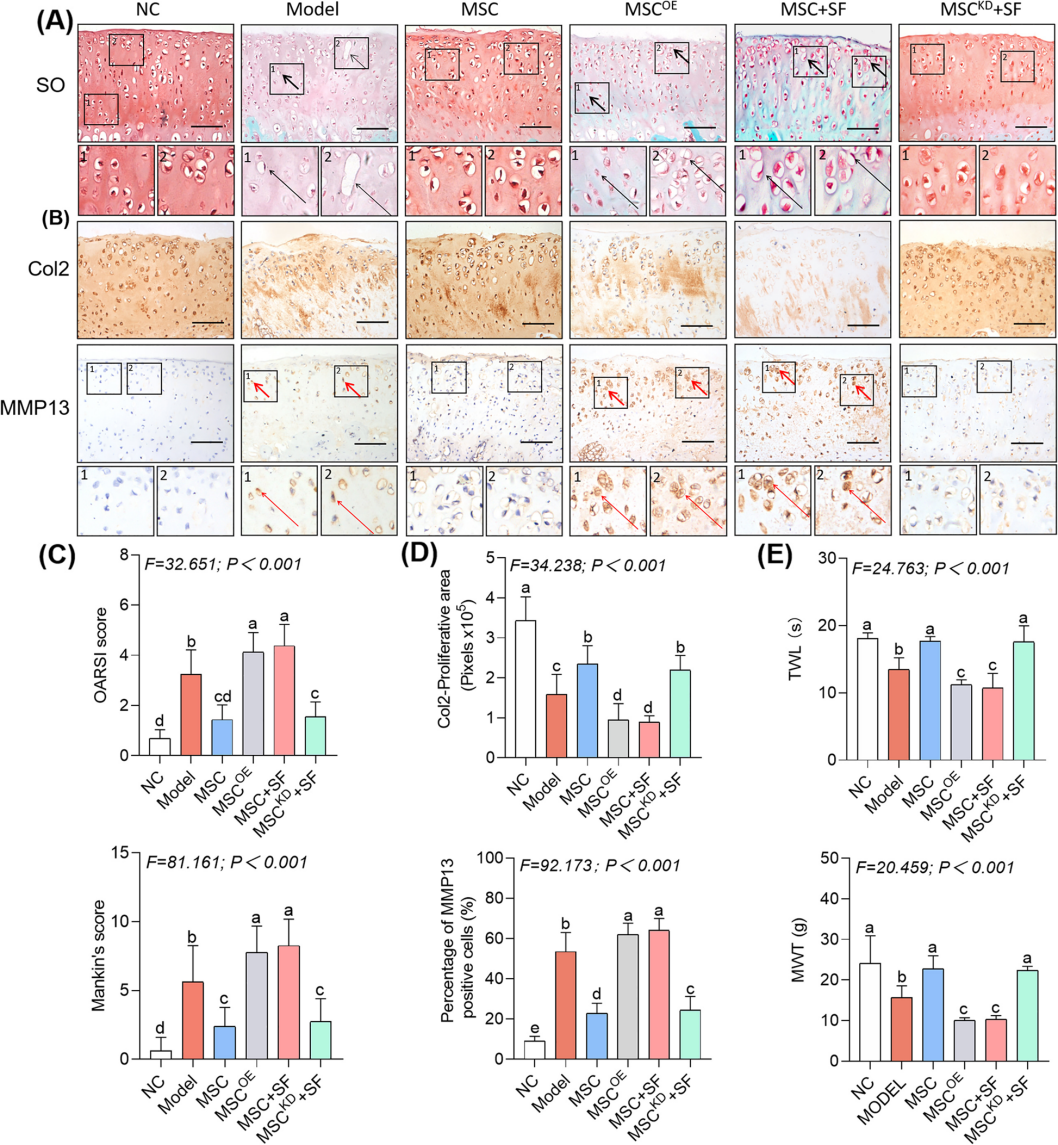
Histopathological and IHC analyses and pain behavior tests in kOA rats treated with normal hucMSCs, hucMSCs with MMP13 overexpression, SF-stimulated hucMSCs and SF-stimulated knockdown MMP13-hucMSCs. **A** Safranin O and Fast green staining of the cartilage, with black arrows indicating hypertrophy or loss of chondrocytes. Scale bars = 50 μm. **B** IHC staining of Col2 and MMP13 on cartilage, with red arrows indicating positive cells. Scale bars = 50 μm. **C** OARSI and Mankin’s scoring of histopathology. **D** Quantitative measurements of the percentage of Col2-positive area and MMP13-positive cells. **E** Measurement of MWT and TWL of rats. Data are expressed as mean ± SD. The different letter symbols (a, b, c, cd, d, and e) indicate significant difference between each other (Fisher*'*s LSD, *P* < 0.05 or *P* < 0.01) in descending order of data from a to z. Although the overlapped letters (c versus cd, and cd versus d) were statistically different, their difference between each other was not significant. All experiments were repeated at least three times

By utilizing the ACLT kOA model, the causative role of MMP13 in hucMSCs-based deterioration of kOA was validated in vivo. As shown in Fig. 5A and C, the kOA cartilage degeneration was not only halted but actually worsened in the MSC^OE^ group (each *P* < 0.01 versus Model scores or versus MSC scores) and the MSC + SF group (each *P* < 0.01 versus Model scores or versus MSC scores). Little difference was seen between the MSC^OE^ group and the MSC + SF group. Inversely, the cartilage degeneration in the MSC^KD^ + SF group was recovered (each *P* < 0.01 versus Model scores), similar to that seen in the MSC group (each *P* > 0.05 versus MSC scores). The ascending order of Mankin’s score and OARSI score were similar and presented as follows: NC < MSC ≈ MSC^KD^ + SF < Model < MSC^OE^ ≈ MSC + SF. As shown in Fig. 5B and D, IHC analysis showed that cartilage anabolic (Col2) and catabolic (MMP13) expressions in the model group and the MSC group were consistent with the above data (Fig. 1B and D). However, the kOA-like abnormalities of Col2 and MMP13 expressions remained significant in the MSC^OE^ group (each *P* < 0.01 versus NC or versus MSC) and the MSC + SF group (each *P* < 0.01 versus NC or versus MSC), which became worse than the model levels (each *P* < 0.01 versus Model for both MSC^OE^ and MSC + SF). As expected, these abnormalities were dramatically restored in the MSC^KD^ + SF group (each *P* < 0.01 versus Model), and the expressions of Col2 and MMP13 reached levels similar to that in the MSC group (each *P* > 0.05 versus MSC). As illustrated in Fig. 5E, the pain behavior tests resulted in a similar tendency, in which the MWT and TWL levels were significantly decreased in the following order: NC > MSC ≈ MSC^KD^ + SF > Model > MSC^OE^ ≈ MSC + SF. The above data verifies that MMP13 overexpression not only counteracted the anti-kOA efficacy of hucMSCs but also deteriorated the cartilage degeneration and pain. Therefore, MMP13 acts as the key determinant of the effects of hucMSC-based therapy and its anti-kOA effects when its levels are low and pro-kOA when its levels are high.

### A MAPKs-AP1 signaling pathway mediates MMP13 overexpression in hucMSCs under an AIM

**Fig. 6:**
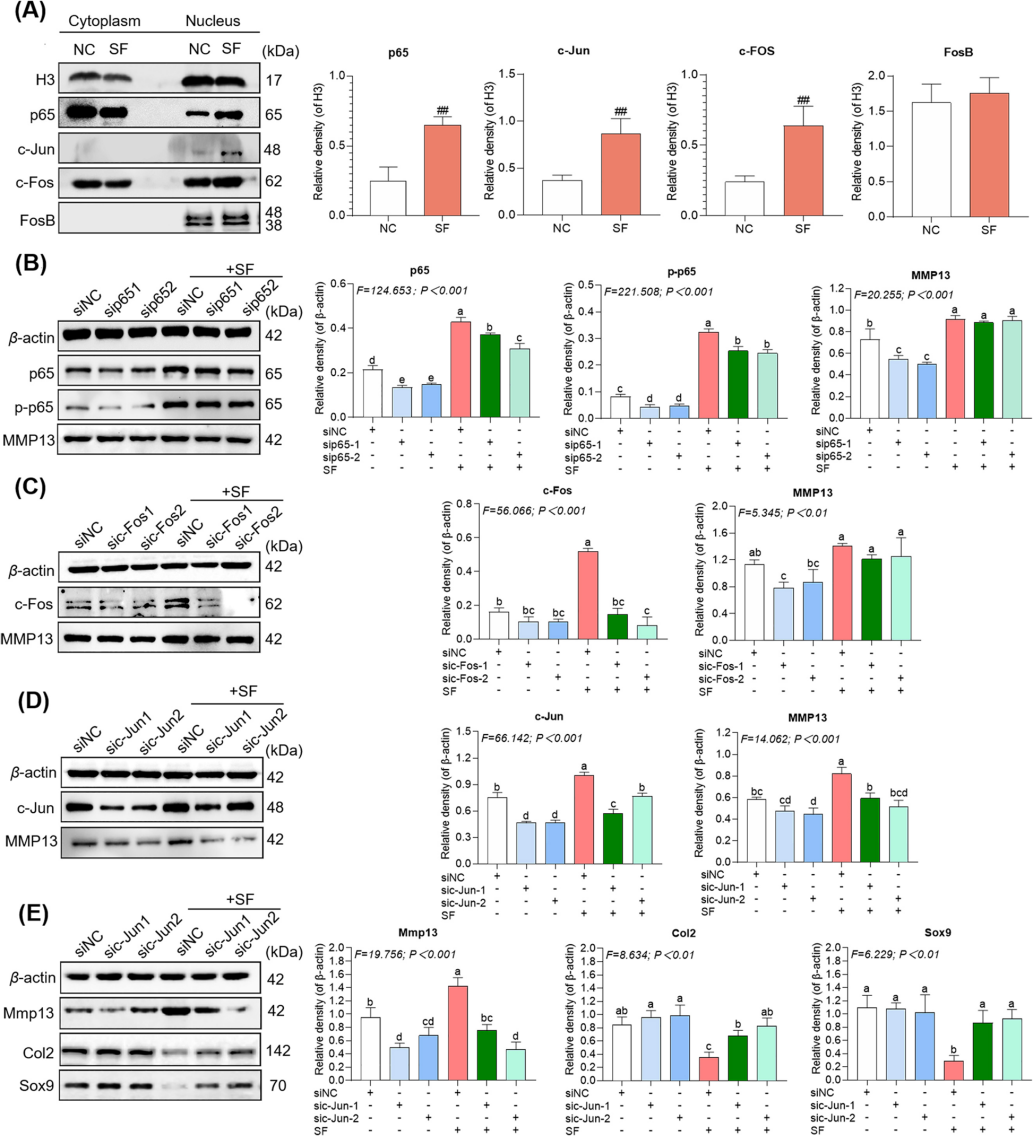
SF-mediated transcription factors upregulate MMP13. **A** Expression of hucMSC transcription factors treated with SF. **B** Expression of hucMSCs treated with SF and p65-SiRNA. **C** Expression of hucMSCs treated with SF and c-Fos-SiRNA. **D** Expression of hucMSCs treated with SF and c-Jun-SiRNA. **E** Expression of chondrocytes treated with hucMSCs (with c-Jun knockdown) and SF. Values are presented as mean ± SD. The different letter symbols (a, ab, b, bc, c, cd, bcd, d, and e) indicate significant difference between each other (Fisher*'*s LSD, *P* < 0.05 or *P* < 0.01) in descending order of data from a to z, in which the overlapped letters (e.g., b versus bc, c versus cd, and cd versus d) were statistically but not significantly differed. All experiments were repeated at least three times

The transcription factors (p65, c-Jun, c-Fos, and FosB) for MMP13 and the corresponding upstream signaling molecules were analyzed to clarify the MMP13-dependent mechanism by which an AIM negatively effects hucMSCs-based therapy for kOA. As illustrated in Fig. 6A, the nuclear expressions of c-Jun, c-Fos, and p65 in hucMSCs were upregulated significantly in the presence of SF (each *P* < 0.01 versus NC), while little change in FosB was seen (*P* > 0.05 versus NC), suggesting that p65, c-Jun, and c-Fos participated in the overexpression of MMP13 in hucMSCs in the presence of SF. For validation, p65-siRNA, c-Jun-siRNA, and c-Fos-siRNA were used to knockdown these factors. As illustrated in Fig. 6B to D, only c-Jun-knockdown (si c-Jun-1 group and si c-Jun-2 group) significantly downregulated MMP13 expression of hucMSCs in the presence of SF (each *P* < 0.01 versus si NC + SF), which produced levels of MMP13 similar to that seen in the absence of SF (*P* > 0.05 versus si NC). Moreover, as illustrated in Fig. 6E, the c-Jun-knockdown hucMSCs significantly reversed the abnormal expression levels of Col2 and Sox9 and MMP13 of chondrocytes induced by hucMSCs in the presence of SF (each *P* < 0.01 versus si NC + SF), resulting in normalization of the anabolism and catabolism states of chondrocytes (*P* > 0.05 versus si NC). These findings suggested that c-Jun (AP1) is the key transcription factor by which SF exposure regulates the expression of MMP13 in hucMSCs.

**Fig. 7:**
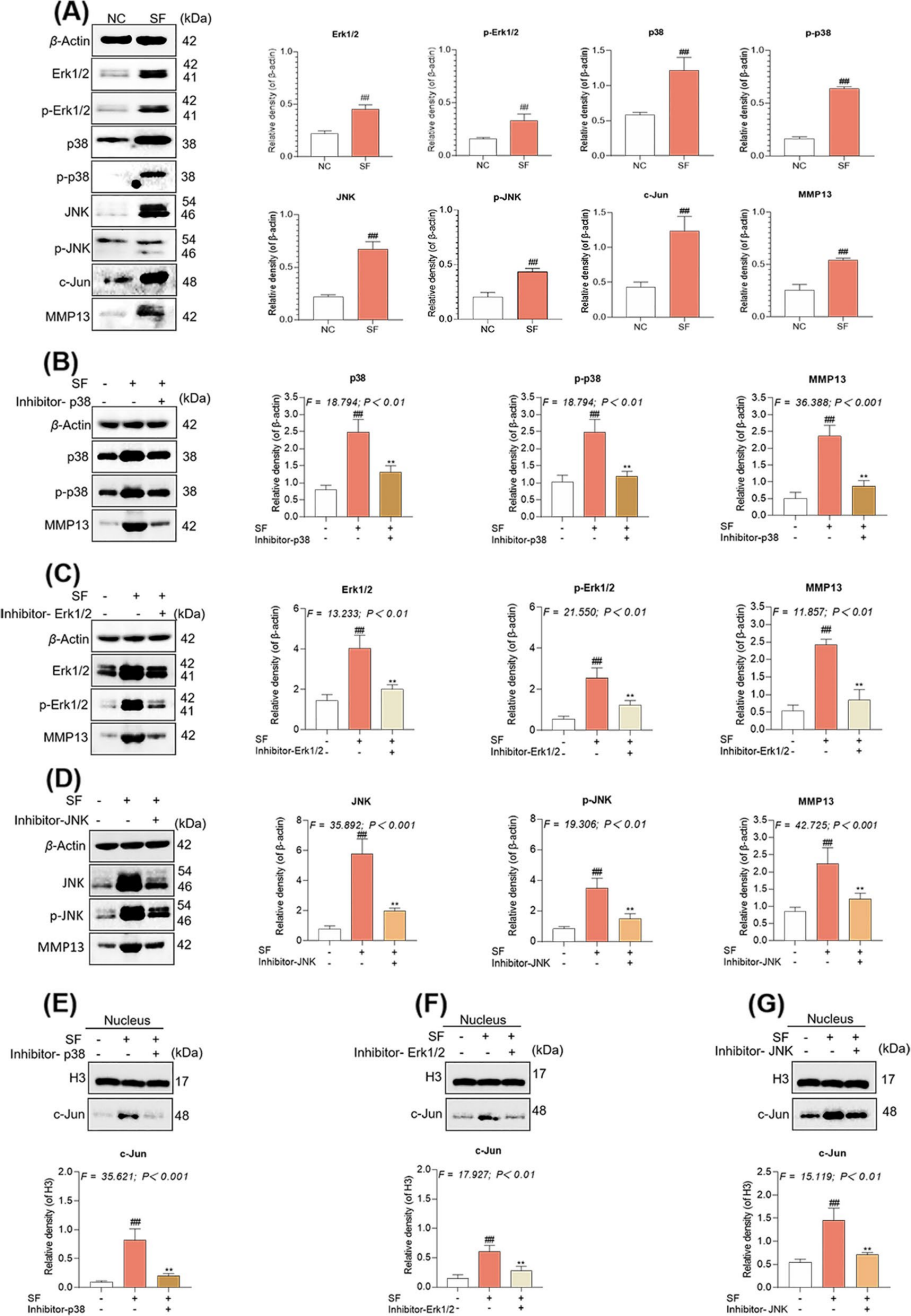
SF upregulates MMP13 expression in hucMSCs via a MAPK-AP1 pathway. **A** Protein bands and quantitation of their expression in hucMSCs treated with SF. **B** hucMSCs treated with SF and p38 inhibitor. **C** hucMSCs treated with SF and Erk1/2 inhibitor. **D** hucMSCs treated with SF and JNK inhibitor. **E** hucMSCs nuclei treated with SF and p38 inhibitor. **F** hucMSCs nuclei treated with SF and Erk1/2 inhibitor. **G** hucMSCs nuclei treated with SF and JNK inhibitor. Values are presented as mean ± SD. ^#^*P* < 0.05 and ^##^*P* < 0.01 versus NC group; ^*^P < 0.05 and ^**^P < 0.01 versus SF group. All experiments were repeated at least three times

Accordingly, the upstream pathway (MAPKs) of c-Jun was analyzed. As illustrated in Fig. 7A, SF activated the MAPK family members, p38, JNK, and Erk1/2, through overexpression and phosphorylation (each *P* < 0.01 versus NC). As illustrated in Fig. 7B to D, by using inhibitors of p38 (SB203580), Erk1/2 (U0126-EtOH), and JNK (SP60025), the effect of SF on the overexpression of MMP13 in hucMSCs was significantly suppressed (each *P* < 0.01 versus SF). As illustrated in Fig. 7E to G, the intranuclear expression of c-Jun was significantly inhibited by these inhibitors (each *P* < 0.01 versus SF), suggesting a direct and independent regulatory role of p38, JNK, and Erk1/2 on c-Jun in the presence of SF.

## Discussion

In a healthy joint cavity, SF is produced by the synovial membrane and acts as a medium for transporting nutrients, enzymes, cytokines, and growth factors to cartilage [48, 49]. However, once synovitis develops, it induces effusion of “inflamed” SF rich in inflammatory factors and thereby results in an AIM and aggravation of kOA progression [50–52]. A large number of inflammatory factors, such as IL-1, IL-6, IL-8, IL-17, and TNF-α, have been detected in SF samples from patients with kOA, and it closely correlates with the severity of kOA symptoms [28, 53–56]. Interestingly, native (not culture-expanded) joint-resident MSCs are present with SF in healthy joints, and these cells are presumed to be a primary driver of cartilage repair in adulthood, whereas MSCs hardly survive when proinflammatory SF is present in patients with kOA [57, 58]. These findings indicate that effusion of proinflammatory SF may deteriorate not only the joint microenvironment but also the internal living conditions of MSCs. This may explain why there have been conflicting outcomes among clinical studies of MSC-based treatment for kOA [16, 21]. Thus, we hypothesized that the absence or presence of proinflammatory SF in the kOA joint cavity may determine the efficacy of MSC-based therapy for kOA.

**Fig. 8:**
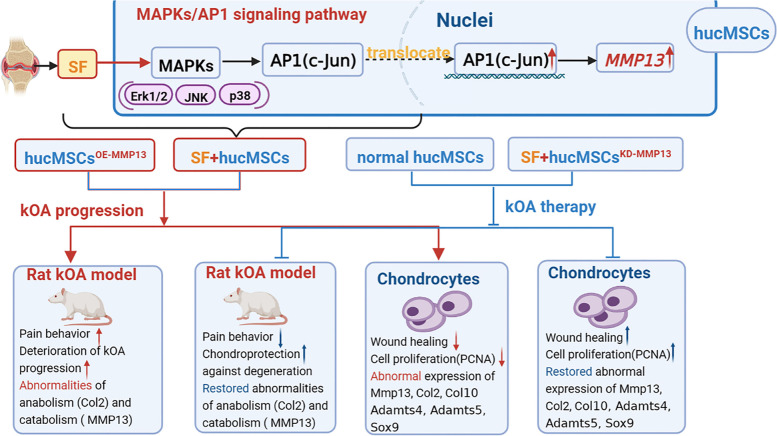
Summary of the impact and underlying mechanism of an inflammatory microenvironment on hucMSCs-based therapy for kOA

Here, we tested this hypothesis and demonstrated the following points: (1) SF from patients with kOA contained a variety of inflammatory factors; (2) hucMSCs could repair the osteoarthritic cartilage, but the chondroprotective effects of hucMSCs were blunted in the presence of SF and instead the cells showed chondrodestructive effects; (3) overexpression of MMP13 was a key and specific effector of this differential effects of hucMSCs; and (4) a MAPKs/AP1 signaling pathway mediated the overexpression of MMP13 in hucMSCs when they were exposed to a SF-based AIM. For the first time, we report evidence that may explain the discrepant effects of MSC-based therapy for kOA and thus why the previous results were inconsistent, as well as providing a mechanism that explains such discrepancies (Fig. 8).

Our findings indicate that MSCs should not be intra-articularly injected to treat kOA when there is evidence of a proinflammatory SF-based microenvironment in the affected joint. The expression levels of MMP13 in the MSCs after injection should be monitored as a biomarker for their efficacy. Moreover, activation of a MAPKs-AP1-MMP13 signaling axis was identified as the underlying mechanism that determines the efficacy of the MSC-based therapy. Thus, either a pharmacological inhibitor of MMP13 (or neutralizing antibody) or one targeting the upstream transcriptional pathway, should be applied to the MSCs before injection occurs, especially if the inflammatory status of the targeted joint cannot be determined.

The MAPK members (e.g., Erk1/2, JNK, and p38) have been reported to participate in kOA development by inhibiting the viability of MSCs in the subchondral bone, by inducing their apoptosis and/or suppressing their differentiation [59, 60]. These kinases can also promote the inflammatory state of MSCs by increasing the expression of proinflammatory factors (e.g., IL-6) and chemokines (e.g., MCP-1) for immune cell recruitment [61]. Furthermore, inhibition of JNK and p38 benefits chondrogenic differentiation of MSCs [61]. As the downstream effector of MAPKs, c-Jun has been reported to regulate the expression of proinflammatory factors (e.g., IL-8) and affect the differentiation of MSCs [62]. Also, MMP13 participates in the hypertrophic process of MSCs, while inhibiting the chondrogenic differentiation of MSCs [63, 64]. Therefore, the MAPKs-AP1-MMP13 axis plays negative roles in the biology of MSCs, and our study here adds further insight into this process while showing that these cells also regulate the expression of MMP13 and thus their catabolic activity.

The AIM can be characterized as a chronic inflammatory condition, since synovial inflammation commonly occurs at the early stage of kOA and the patients always suffered a long history of persistent inflammation [65]. However, the inflammatory stimulation on exogenous MSCs should be acute, since the survival time of MSCs (especially the allogeneic and heterologous MSCs) in recipient body is short [66, 67]. Therefore, in this study, hucMSCs were treated with SF for 24 ~ 48 h to reproduce the acute inflammatory stimulation. Previously, the impact of acute inflammatory microenvironment on MSCs has been reported with inconsistent conclusions [30–32, 68–74]. On the “good” side, some cytokines (e.g., IFNγ, TNFα, IL-1, and IL-10) that exist in the inflammatory microenvironment can stimulate the immunosuppressive activity of MSCs, resulting in restoration of abnormal immune responses [68–74]. For instance, IFN-γ was necessary for the immunosuppression of T cells by MSCs, and blockage of the receptor for IFN-γ or TNFα could impair the immunosuppressive activity of MSCs [73, 75, 76]. On the “bad” side, the cytokines (e.g.*,* IL-1, IL-6, TNFα) in some cases may cause inflammatory damage and impair the function of MSCs [77–79]. Of these, IL-1β could inhibit MSCs’ chondrogenic differentiation and aggravates their inflammatory state by increasing the expression levels of proinflammatory factors (e.g., IL-6 and IL-8) and matrix-degrading enzymes [28, 34, 77, 78, 80, 81]. Furthermore, IL-6 could induce senescence of MSCs, thereby impairing their stemness and functions [79]. While TNF-α might not inhibit the chondrogenic differentiation of MSCs as strongly as IL-1, it could impair their chondrogenesis at the onset or during progression of differentiation [77]. Kim et al. [33] concluded that acute inflammation might benefit the immunosuppressive activity of MSCs, while chronic inflammation might impair these cells, suggesting that different conditions of the inflammatory microenvironment (acute vs. chronic or mild vs. severe) might determine the different outcomes of MSCs. That study explained why previous studies reported the inconsistent impact of an inflammatory microenvironment on MSCs.

It may be possible that the injection of human samples (MSCs or SF) into rat joints might cause a heterogeneous allergic response or immune rejection and thereby interferes with the interpretation of our animal experiments. However, we have previous utilized not only articular but also intravenous injection of human MSCs (hucMSCs and adipose-derived stem cells, ADSCs) to treat rats and obtained positive outcomes without any immune rejection or adverse events [44–46]. In this study, we also found no adverse reaction in rats after the injection, indicating only therapeutic efficacy other than interference from heterologous hucMSCs. Although the human SF contained proinflammatory factors that could trigger immune responses, neither heterogeneous allergy/rejection reaction nor deterioration of cartilage destruction/pain was observed after SF injection in our assays. Therefore, we believe that these concerns are not valid in this case and that our approach offers a feasible methodology and that our results are well controlled and convincing. Even so, as many different inflammatory factors contribute to the SF-mediated AIM, each factor should be individually tested in the future to determine their contribution to the negative effects on MSC-based therapy.

## Conclusions

This study demonstrated that an articular injection of hucMSCs exerted pain-relieving and cartilage-repairing efficacy in a kOA rat model and restored a more normal profile of anabolism and catabolism of chondrocytes. However, in the presence of “inflamed” SF (i.e., an AIM condition), the chondroprotective effects of hucMSCs were blunted and such cellular-based therapy actually became chondrodestructive, significantly worsening kOA progression in this model. Overexpression of MMP13, downstream of a MAPKs-AP1 signaling axis, in hucMSCs was found to be the key and specific effector of this outcome. For the first time, to the best of our knowledge, we demonstrated the cellular and molecular mechanisms that explains the discrepant results of MSC-based therapy on kOA. Furthermore, these results suggest that the inflammatory status of the affected joint should be assessed prior to treatment or that the MMP13 pathway should be targeted in the MSCs before injection.

## Supplementary Information

**Additional file 1:  Figures S1. **The design flowchart for this study.

**Additional file 2: Table S1.** Information of antibodies.

**Additional file 3: Figure S2.** The design flowchart for in vivo efficacy evaluation of hucMSCs in the presence/absence of AIM.

**Additional file 4: Figure S3.** The design flowchart for in vitro evaluation of the impact of AIM on the state of hucMSCs.

**Additional file 5: Figure S4.** The design flowchart for in vitro evaluation of the impact of AIM on the function of hucMSCs.

**Additional file 6: Table S2.** The sequences of siRNA.

**Additional file 7: Figure S5.** The design flowchart for in vitro determination of the role of MMP13 in hucMSCs under an AIM.

**Additional file 8: Figure S6.** The design flowchart for in vivo determination of the role of MMP13 in hucMSCs under an AIM.

**Additional file 9: Table S3.** The inflammatory factors in the mixed SF.

**Additional file 10: Figure S7.** Surface marker identification of hucMSCs by flow cytometry.

## Data Availability

The datasets used and/or analyzed during the current study are available from the corresponding author on reasonable request.

## Abbreviations

kOA: Knee osteoarthritis
NSAIDs: Nonsteroidal anti-inflammatory drugs
MSCs: Mesenchymal stem cells
WOMAC: Western Ontario and McMaster Universities Osteoarthritis Index
VAS: Visual Analog Scale
SF: Synovial fluid
IL-1β: Interleukin-1β
IFN-γ: Interferon-gamma
TNF-α: Tumor necrosis factor-α
AIM: Inflammatory microenvironment
MMP13: Matrix metalloproteinase 13
BCA: Bicinchoninic acid
DAPI: 4',6-Diamidino-2-phenylindole
TBS: Tris-buffered saline
BSA: Bovine serum albumin
PBS: Phosphate-buffered saline
IHC: Immunohistochemistry
ACLT: Anterior cruciate ligament transection
MWT: Mechanical withdrawal threshold
TWL: Thermal withdrawal latency
PFA: Paraformaldehyde
EDTA: Ethylene-diamine tetra acetic acid
OARSI: Osteoarthritis Research Society International
qRT-PCR: Quantitative real-time PCR
WB: Western blot
LSD: Least significant difference
DPS: Data Processing System
ADSCs: Adipose-derived stem cells
