# Supplementary figures and images for: Detrimental alteration of mesenchymal stem cells by an articular inflammatory microenvironment results in deterioration of osteoarthritis
Source: BMC Med. 2023 Jun 19;21:215. doi: 10.1186/s12916-023-02923-6 (PMC10280917; doi:10.1186/s12916-023-02923-6)

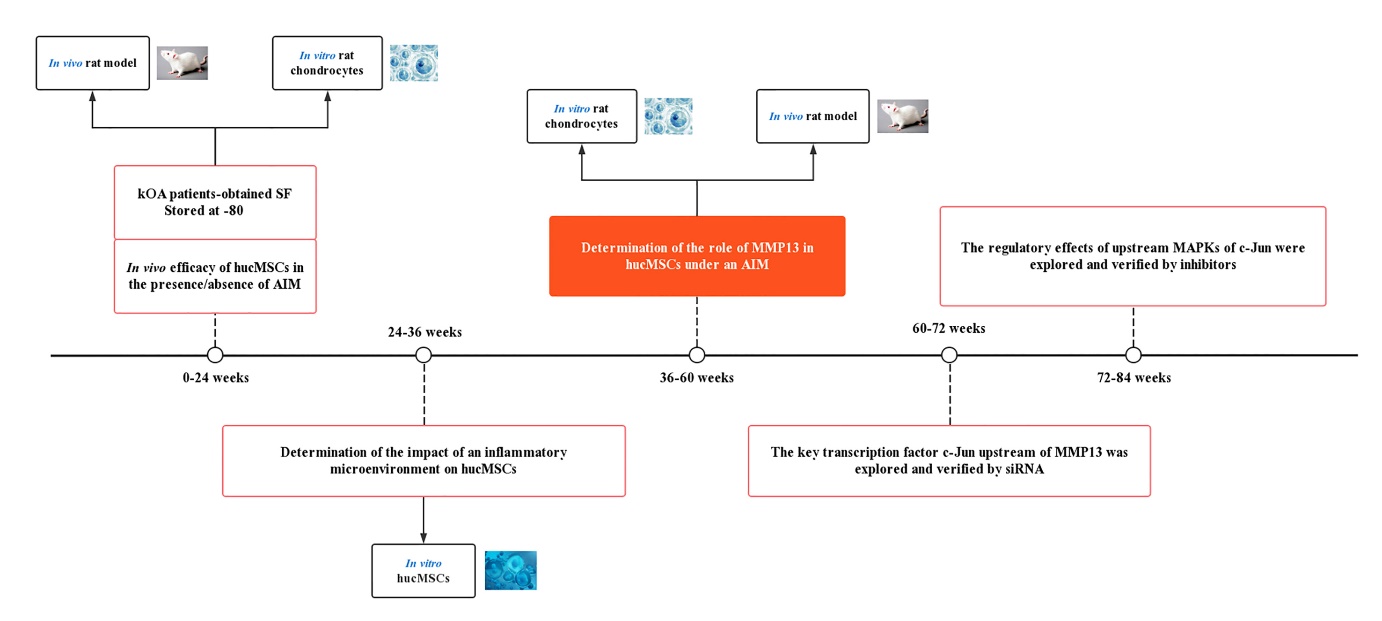


Figure S1. The design flowchart for this study.

Supplement: Supplementary file 1 — Additional file 1: Figures S1. The design flowchart for this study. [file 12916_2023_2923_MOESM1_ESM.docx]

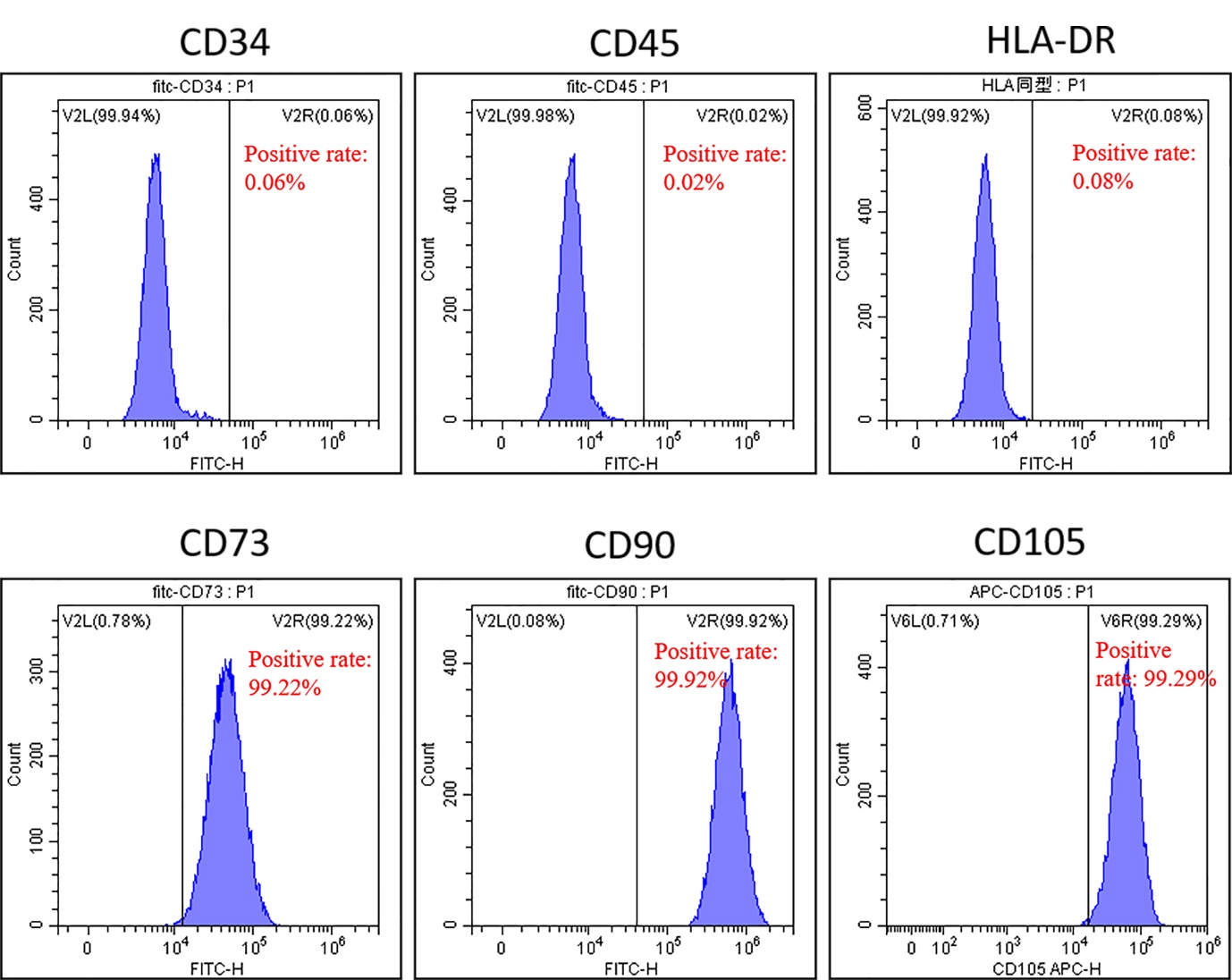


Figure S7. Surface marker identification of hucMSCs by flow cytometry.

Supplement: Supplementary file 10 — Additional file 10: Figure S7. Surface marker identification of hucMSCs by flow cytometry. [file 12916_2023_2923_MOESM10_ESM.docx]
