# Supplementary material for: Detrimental alteration of mesenchymal stem cells by an articular inflammatory microenvironment results in deterioration of osteoarthritis
Source: BMC Med. 2023 Jun 19;21:215. doi: 10.1186/s12916-023-02923-6 (PMC10280917; doi:10.1186/s12916-023-02923-6)
Supplement: Supplementary file 2 — Additional file 2: Table S1. Information of antibodies. [file 12916_2023_2923_MOESM2_ESM.docx]

Table S1. Information of antibodies

| Targets | Manufacturer | Catalog Number | Applications | Dilutions |
| --- | --- | --- | --- | --- |
| Collagen II | Abcam | ab34712 | IHC | 1:200 |
| MMP13 | Abcam | ab39012 | IHC | 1:200 |
| *β*-actin | Novus | NB600-501 | WB | 1:5000 |
| Nanog | CST | 4903S | WB | 1:2000 |
| Sox2 | CST | 3579S | WB | 1:1000 |
| Oct4 | CST | 2750S | WB | 1:1000 |
| Sall4 | CST | 5850S | WB | 1:1000 |
| PCNA | CST | 13110S | IF | 1:400 |
| MMP13 | Novus | NBP2-45887 | WB | 1:4000 |
| Adamts4 | Abcam | ab185722 | WB | 1:1000 |
| Collagen II | Abcam | ab34712 | WB | 1:1000 |
| Collagen X | Abcam | ab58632 | WB | 1:200 |
| FITC anti-human CD34 | BioLegend | 343504 | Flow cytometry | / |
| FITC anti-human CD45 | BioLegend | 304006 | Flow cytometry | / |
| FITC anti-human HLA-DR | BioLegend | 307604 | Flow cytometry | / |
| FITC anti-human CD73 | BioLegend | 344016 | Flow cytometry | / |
| FITC anti-human CD90 | BioLegend | 328108 | Flow cytometry | / |
| APC-anto-human-CD105 | BioLegend | 800508 | Flow cytometry | / |
| p65 | CST | 8242S | WB | 1:1000 |
| p-p65 | CST | 3033S | WB | 1:1000 |
| c-Jun | CST | 9165S | WB | 1:1000 |
| c-Fos | CST | 4384T | WB | 1:1000 |
| FosB | CST | 2263S | WB | 1:1000 |
| Sox9 | Abcam | ab185230 | WB | 1:1000 |
| Erk1/2 | CST | 4695S | WB | 1:1000 |
| p-Erk1/2 | CST | 4370T | WB | 1:2000 |
| p38 | CST | 9212S | WB | 1:1000 |
| p-p38 | CST | 4511S | WB | 1:1000 |
| JNK | CST | 9252S | WB | 1:2000 |
| p-JNK | CST | 9255S | WB | 1:2000 |
| Histone H3 | CST | 4499S | WB | 1:2000 |
| Horseradish Peroxidase conjugated Goat Anti-Mouse IgG (H+L) | JIR | 115-035-062 | WB | 1:5,000 |
| Horseradish Peroxidase conjugated Goat Anti-Rabbit IgG (H+L) | JIR | 111-035-045 | WB | 1:5,000 |

Abcam: Abcam (Cambridge, UK), Novus: Novus (Colorado, USA), CST: Cell Signaling Technology Inc. (MA, USA), BioLegend: BioLegend (Californa, USA), JIR: Jackson ImmunoResearch Laboratories Inc. (Pennsylvania, USA), IHC: Immunohistochemistry, WB: western-blot, IF: Immunofluorescence
