# Supplementary material for: Detrimental alteration of mesenchymal stem cells by an articular inflammatory microenvironment results in deterioration of osteoarthritis
Source: BMC Med. 2023 Jun 19;21:215. doi: 10.1186/s12916-023-02923-6 (PMC10280917; doi:10.1186/s12916-023-02923-6)
Supplement: Supplementary file 5 — Additional file 5: Figure S4. The design flowchart for in vitro evaluation of the impact of AIM on the function of hucMSCs. [file 12916_2023_2923_MOESM5_ESM.docx]

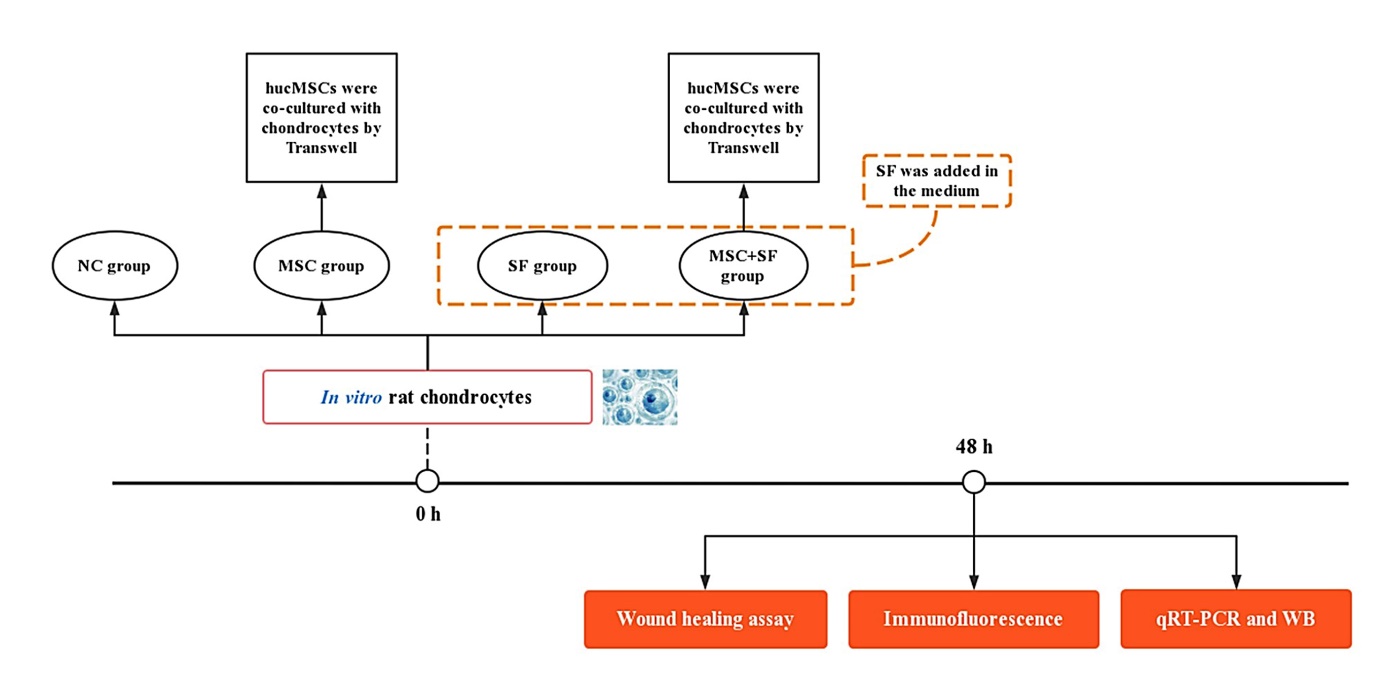


Figure S4. The design flowchart for *in vitro* evaluation of the impact of AIM on the function of hucMSCs.
