# Supplementary material for: Detrimental alteration of mesenchymal stem cells by an articular inflammatory microenvironment results in deterioration of osteoarthritis
Source: BMC Med. 2023 Jun 19;21:215. doi: 10.1186/s12916-023-02923-6 (PMC10280917; doi:10.1186/s12916-023-02923-6)
Supplement: Supplementary file 6 — Additional file 6: Table S2. The sequences of siRNA. [file 12916_2023_2923_MOESM6_ESM.docx]

Table S2. The sequences of siRNA

| Gene (Human) | Sense | Anti-sense |
| --- | --- | --- |
| MMP13-siRNA | 5′-AACGAAAUAUCAAAGUCAUUA-3′ | 5′-UAAUGACUUUGAUAUUUCGUU-3′ |
| p65-siRNA-1 | 5′-GCGACAAGGUGCAGAAAGATT-3′ | 5′-UCUUUCUGCACCUUGUCGCTT-3′ |
| p65-siRNA-2 | 5′-GCCCUAUCCCUUUACGUCATT-3′ | 5′-UGACGUAAAGGGAUAGGGCTT-3′ |
| c-Fos-siRNA-1 | 5′-GCAAUAGUGUGUUCUGAUUTT-3′ | 5′-AAUCAGAACACACUAUUGCTT-3′ |
| c-Fos-siRNA-2 | 5′-GAAUCCGAAGGGAAAGGAATT-3′ | 5′-UUCCUUUCCCUUCGGAUUCTT-3′ |
| c-Jun-siRNA-1 | 5′-GUCAUGAACCACGUUAACATT-3′ | 5′-UGUUAACGUGGUUCAUGACTT-3′ |
| c-Jun-siRNA-2 | 5′-ACGCAAACCUCAGCAACUUTT-3′ | 5′-AAGUUGCUGAGGUUUGCGUTT-3′ |
