# Supplementary material for: Detrimental alteration of mesenchymal stem cells by an articular inflammatory microenvironment results in deterioration of osteoarthritis
Source: BMC Med. 2023 Jun 19;21:215. doi: 10.1186/s12916-023-02923-6 (PMC10280917; doi:10.1186/s12916-023-02923-6)
Supplement: Supplementary file 9 — Additional file 9: Table S3. The inflammatory factors in the mixed SF. [file 12916_2023_2923_MOESM9_ESM.docx]

Table S3. The inflammatory factors in the mixed SF

| Inflammatory factors in the mixed SF | MFI | Concentrations (pg/ml) |
| --- | --- | --- |
| IL-1α | 21 | 2.55 |
| IL-1β | 19 | 0.25 |
| IL-6 | 837 | 104.79 |
| IL-8 | 68 | 6.63 |
| IL-12p40 | 21 | 5.08 |
| IL-17 | 19 | 1.45 |
| TNFα | 26 | 3.84 |
| TNFβ | 59 | 7.31 |
| IFNγ | 35 | 12.47 |

MFI: Median Fluorescence Intensity
